# Efficient and Non-Invasive Grading of Chinese Mitten Crab Based on Fatness Estimated by Combing Machine Vision and Deep Learning

**DOI:** 10.3390/foods14111989

**Published:** 2025-06-05

**Authors:** Jiangtao Li, Hongbao Ye, Chengquan Zhou, Xiaolian Yang, Zhuo Li, Qiquan Wei, Chen Li, Dawei Sun

**Affiliations:** 1 Huzhou Academy of Agricultural Sciences, Huzhou 313000, China; 2 Institute of Agricultural Equipment, Zhejiang Academy of Agricultural Sciences, Hangzhou 310021, China; 3 Key Laboratory of Agricultural Equipment for Hilly and Mountainous Areas in Southeastern China (Co-Construction by Ministry and Province), Ministry of Agriculture and Rural Affairs, Hangzhou 310021, China

**Keywords:** Chinese mitten crab, *Eriocheir sinensis*, grading, machine learning, YOLO

## Abstract

The Chinese mitten crab (*Eriocheir sinensis*) is a high-value seafood. Efficient quality-grading methods are needed to meet rapid increases in demand. The current grading system for crabs primarily relies on manual observations and weights; it is thus inefficient, requires large amounts of labor, is costly, and no longer meets the requirements for the market. Here, we employed computer vision techniques combined with deep learning modeling to efficiently quantify key physiological traits, such as sex identification, carapace dimensions (length and width), and fatness assessment for quality classification. To this end, a YOLOv5-seg integrated with an SE attention model was developed using 2282 RGB images and manual measurements of the physiological traits of 300 crabs. The RGB dataset was further augmented by rotating and resizing. The results revealed that the accuracy of sex recognition was 100%, and the mAP for carapace segmentation was 0.995, which was superior to YOLOv8-seg and other variants. In addition, we proposed an improved conditional factor K to evaluate the fatness of crabs and classify their quality based on fatness. The consistency between the grading method proposed in this article and manual grading was 100%. This study could aid in developing precise and non-destructive grading systems for the aquaculture and food industries.

## 1. Introduction

The Chinese mitten crab (*Eriocheir sinensis*) is a highly valued aquatic product in China for its tender meat, high protein content, diverse vitamins, trace elements, and distinctive flavor [1,2,3]. It thus holds an economically important position in China as a high-quality aquaculture product. The Chinese mitten crab is extensively farmed across China, with its aquaculture range spanning from the Liao River basin in the northeast to the Pearl River delta in the south [4,5]. China’s aquaculture sector yielded approximately 775,900 metric tons of Chinese mitten crab, generating an estimated market value exceeding 100 billion Yuan [6]. As living standards and purchasing power continue to rise, consumer demand has shifted toward premium aquatic products, such as Chinese mitten crabs, with parallel increases in quality requirements across supply chains [7,8]. Consequently, rapid, precise, and non-invasive assessment of mitten crab quality is crucial for satisfying diverse consumer demands, facilitating tiered marketing and boosting crab farmers’ revenue.

Grading systems significantly enhance the utilization and economic value of agricultural products, as standardized classifications help meet diverse consumer demands and permit premium pricing [9]. Aquatic products are high-value agricultural commodities that require particularly rigorous grading. Current industrial standards for crab grading prioritize three measurable traits: carapace width (size), live weight, and sexual dimorphism. These parameters form the basis of traditional quality classification, while fatness remains an important but less standardized quality determinant [10,11,12]. Effective grading not only increases the product value but also standardizes supply chains, mitigates fraudulent practices, and strengthens consumer trust [13]. Thus, establishing a biologically informed and market-driven grading system for aquatic products is crucial for promoting the sustainable development of the agricultural industry.

Agricultural product grading predominantly utilizes two approaches: manual observation and automated grading [14]. Manual grading methods, though labor-intensive and subjective, offer advantages such as low initial cost, operational simplicity, and no requirement for specialized equipment or technical training. These features make manual grading accessible and practical in small-scale aquaculture operations or regions with limited resources [15]. The conventional manual assessment methods for crab grading present three fundamental limitations: (1) high labor requirements resulting in low throughput capacity, (2) operator-dependent subjectivity causing inconsistent evaluations, and (3) processing speeds incompatible with contemporary industrial scaling needs. These constraints ultimately hinder the rapid development of the modern crab market [16]. Modern sensing technologies, particularly machine vision systems, hyperspectral imaging, and multimodal sensor arrays, have emerged as transformative solutions for automated quality assessment for aquaculture products, particularly fish and shrimp. For instance, spectroscopic techniques such as near-infrared (NIR) and hyperspectral imaging have been applied to evaluate internal quality attributes such as freshness and fat content in fish fillets [17] and shrimp [18]. Previous research has explored the use of imaging and machine learning for classifying mussels based on inner shell color features [19]. These digital analytical methods enable rapid, objective, and high-throughput evaluation of critical quality parameters, overcoming the limitations of conventional manual approaches [20].

Machine vision systems have become increasingly prevalent in aquatic product processing due to their non-invasive nature, operational efficiency, and cost-effectiveness. Compared with spectroscopy, which primarily provides internal or compositional information such as moisture, fat, or protein content, machine vision is more practical for real-time, non-destructive, and low-cost morphological assessments. Additionally, machine vision systems can be more easily integrated into automated online grading devices due to their faster data acquisition and processing speeds, as well as the availability of lightweight and cost-effective imaging hardware [15,21,22]. Therefore, this technology enables high-speed, objective quality evaluation while preserving product integrity, making it particularly valuable for industrial-scale grading applications. Feng et al. developed a machine-vision-based algorithm for identifying river crabs by extracting multi-scale and deep features from crab exoskeleton images; it thus offers a theoretical foundation for crab traceability [23]. Shan et al. utilized machine vision technology to develop a model using projection images from both sides of the silver pomfret to evaluate its quality [24]. Tang et al. implemented a deep-learning-based approach for the morphometric analysis of mantis shrimp, achieving measurement errors of 1.74% for total carapace width and 3.33% for carapace length when quantifying projected area, total carapace width, and carapace length parameters [25]. Notably, the model achieved a mean absolute percentage error (MAPE) of 2.23% in predicting mantis shrimp body weight, demonstrating the efficacy of deep learning in crustacean morphometrics. Although computer vision is widely used in the seafood industry, research on its application for grading mitten crab quality remains limited.

YOLOv5 is a widely used, high-speed, and accurate object detection algorithm known for its robustness in real-time applications. While YOLOv5 is designed for object detection using bounding boxes, YOLOv5s-seg extends this architecture by adding a segmentation branch that can output object masks, allowing for precise contour extraction [26]. This is essential for calculating shape-dependent metrics like carapace area and fatness. Additionally, we further improved YOLOv5s-seg by integrating a squeeze-and-excitation (SE) attention module to enhance the network’s focus on informative features, boosting performance in detecting subtle morphological variations. This enhanced version is referred to as YOLOv5s-seg+SE throughout the manuscript, while YOLOv5s-seg refers to the base model without the SE module in this study for clarity. Eventually, by combining machine vision technology with the YOLOv5s-seg deep learning algorithm, we established detection and segmentation models for identifying crab integrity, sex, and carapace features. Furthermore, we calculated fatness by combining weight and carapace dimension data. Our grading method for Chinese mitten crabs is rapid and non-destructive and combines multiple types of information.

## 2. Materials and Methods

### 2.1. Collection and Preparation of Experimental Samples

**Figure 1:**
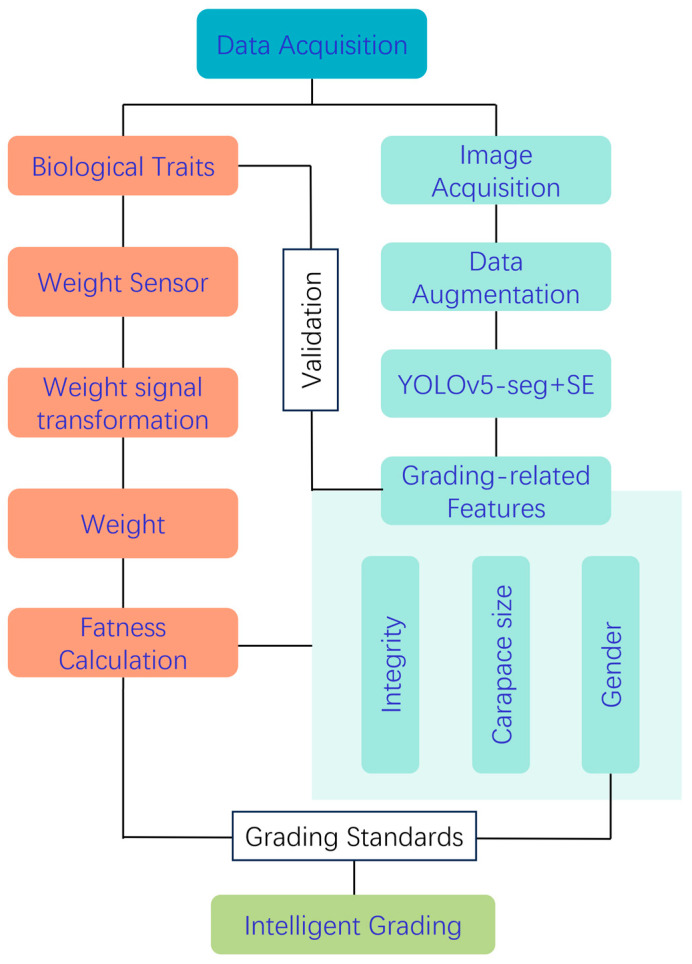
The flowchart of the experimental design for the efficient and non-invasive grading of Chinese mitten crab. The complete process from sample acquisition to final grading, including image capture, sex and integrity detection, carapace feature extraction, fatness calculation based on improved condition factor K, and the final quality classification considering the grading standards.

The experimental samples consisted of 300 crabs from three major crab vendors, including 150 crabs of each sex, which has been described in a previous conference paper [27]. The use of crabs in this study was approved by the IACUC of ZAAS animal welfare community (25ZALAS24). The initial morphometric characterization was performed through the manual measurement of all crab specimens to collect key phenotypic parameters, including umbilical morphology for sexual dimorphism analysis, wet body weight, and carapace dimensions. These ground truth data served as both training inputs and validation benchmarks for the target algorithm. Subsequently, the YOLOv5s-seg architecture enhanced with SE attention mechanisms was employed for automated carapace segmentation, enabling the precise prediction of four critical morphometric features: maximum carapace length, width, thickness, and two-dimensional projected area. These parameters were then employed for fatness, integrity, sex, and size evaluation, which were comprehensively combined for grading. Individual crab weight data were combined with the projected area to calculate fatness. Finally, a multidimensional quality evaluation system was established by combining sex features and fatness metrics. The flowchart of the experimental design is shown in Figure 1, which illustrates the complete process from sample acquisition to final grading, including image capture, sex and integrity detection, carapace feature extraction, fatness calculation based on improved condition factor K, and the final quality classification considering the grading standards.

#### 2.1.1. Integrity Identification

Missing or broken limbs significantly compromise the appearance quality and commercial value of Chinese mitten crabs. In addition, crabs with leg fractures exhibit weakened vitality and have higher risks of bacterial contamination, posing potential health hazards [28,29]. Consequently, automated identification and removal of such defective crabs are essential for food safety. In this study, we constructed an image dataset comprising both intact and leg-deficient crabs, trained a detection model using this dataset, and evaluated its performance on a test set through comparative analysis with manual inspection results. The leg-deficient crab sample set was created by randomly breaking one or several limbs of crabs.

#### 2.1.2. Sex Identification

Sex is a critical grading criterion, because with similar weights and sizes, female crabs have a much higher price compared with male crabs. The sex of each crab was determined and recorded by manually observing the morphology of the abdominal umbilical region with distinct models for male (pointed flap) and female (rounded flap) crabs. Algorithmic detection results were trained and validated against manual observations.

#### 2.1.3. Fatness Calculation

Fatness serves as a key indicator of meat fullness, which directly influences market grading and consumer preference. Higher fatness levels are associated with crabs that are meatier and more desirable. Therefore, it is important to include fatness as a grading indicator. In this research, the fatness was calculated using an improved Fulton condition factor K [30]. The weight, carapace length, and carapace thickness were used to calculate crab fatness. The condition factors were as follows:

Improved condition factor K:

K = 3W/AH(1)

where W = body weight (g), L = carapace length (cm), H = carapace thickness (cm), and A = carapace area (cm^2^). The deduction process used to derive the modified condition factor was outlined in Supplementary File S1, which involves adjusting for carapace area and thickness to improve the biological relevance of the fatness metric, thereby increasing grading accuracy and consistency with consumer expectations.

### 2.2. RGB Image Acquisition

**Figure 2:**
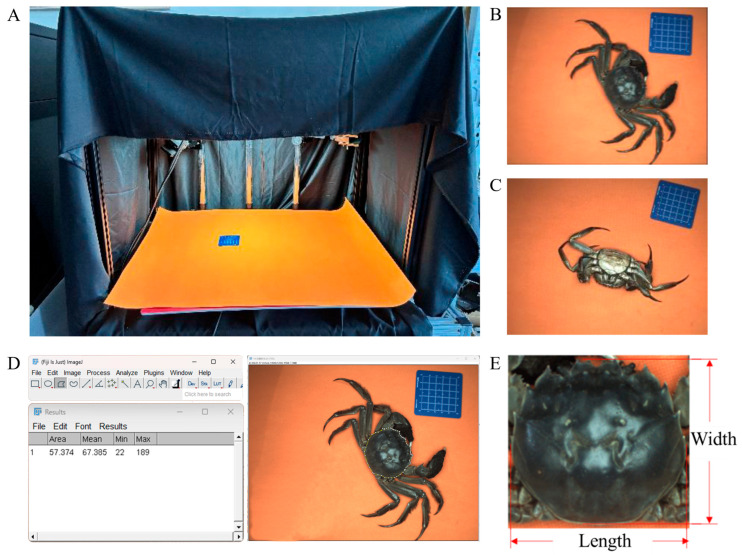
The image acquisition and processing system. (**A**) Image acquisition system; (**B**) dorsal image; (**C**) ventral image; (**D**) region of interest (ROI) identification by manual labeling; (**E**) length and width of carapace.

A custom image acquisition system was developed for the simultaneous capture of morphological and biometric data from crab specimens (Figure 2A). The integrated platform consisted of four core components: (1) an industrial-grade RGB camera (HE020E1GC, Huayong Tech, Shenzhen, China) with 2-megapixel resolution (96 dpi optical precision), which can provide sufficient resolution to accurately capture key morphological features like the carapace and abdominal region while maintaining fast image processing and lower system cost; (2) a high-precision weight sensor (ZNBS-P, Zhongnuo Sensors Corp., Bengbu, China; 0–2 kg range, ±0.01 g accuracy) mounted beneath an orange-colored measurement platform; (3) uniform LED illumination arrays as the white light source; and (4) a programmable control unit for synchronized data acquisition. A computer-based control system processed the collected images and weight data simultaneously.

To minimize the optical interference from ambient lighting and background variability [31,32], the imaging chamber was isolated using light-absorbing black fabric enclosures. Uniform illumination was achieved through a triangular configuration of LED panels (5000 K color temperature) comprising three overhead flanking units above the imaging platform, ensuring consistent spectral distribution across the measurement area. The industrial RGB camera was vertically positioned at an optimal height of 38.5 cm above the measurement platform to acquire the best vision field for carapace imaging. The system underwent calibration checks. A reference scale board was kept within the vision field together with each sample imaging for calibration to maintain measurement accuracy and ensure consistent image quality, as shown in Figure 2. When imaging, each crab was wiped carefully with paper towels to keep the carapace surface dry and clean. Each sample was photographed twice, capturing one dorsal view image (Figure 2B) and one ventral image (Figure 2C), respectively; there was thus a collection of 764 raw images for the image library.

The crabs’ sex was identified through the observation of experienced technicians, and the carapace’s length and width were measured using a vernier caliper. ImageJ software (ver.1.54) was utilized to extract the carapace area. The carapace region, identified as the region of interest (ROI), was manually labeled (Figure 2D). The pixel count within the contour region was determined (Figure 2E). The actual carapace area in cm^2^ was calculated using a standard calibration card included in the image.

### 2.3. Dataset Creation and Image Labeling

**Figure 3:**
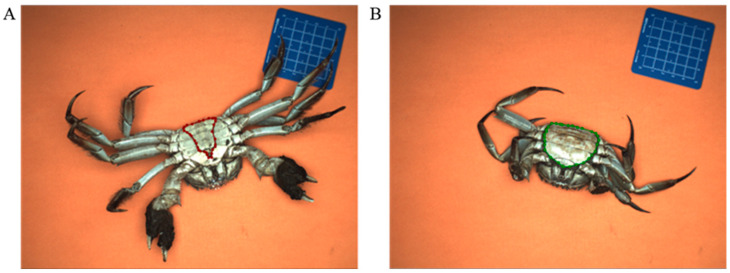
Manual image annotation. (**A**) Manual labeling of male crab feature at the abdominal umbilical region; (**B**) manual labeling of female crab feature at the abdominal umbilical region.

The Labelme tool was utilized to annotate the images by researchers under the supervision of experienced technicians using polygon masking. Manual labeling focused on the umbilical region and carapace of crab specimens (Figure 3). Manual labeling showed low variability, though minor challenges occurred where boundaries were less distinct. Data augmentation methods such as flipping, scaling, and noise were chosen to simulate real-world variability and improve model generalization with approximately half of the final dataset. The primary dataset was established for sexual dimorphism analysis based on distinct umbilical morphologies: males display a triangular, acuminate abdominal flap (Figure 3A), while females present a broader, semicircular structure (Figure 3B). These diagnostically significant features served as the morphological basis for automated gender classification. A similar augmentation process was applied to the second dataset comprising images of crab carapaces. All geometric transformations (e.g., rotation, flipping, scaling) were applied jointly to the images and their corresponding polygon masks to preserve label accuracy. This study employed data augmentation techniques, including SMOTE, rotation, translation, scaling, and shearing, to create an image dataset of 2292 images, equally divided between the umbilical region and carapace.

The complete dataset was partitioned into training (80%), validation (10%), and test (10%) subsets to facilitate model development and evaluation. Dataset splitting was stratified to maintain balanced distributions of sex, size, and integrity status across training, validation, and test sets, ensuring representative model training and evaluation. This stratified division yielded 916 annotated images for algorithm training, with 115 images each reserved for hyperparameter validation and final performance testing, ensuring a statistically robust assessment of both sex identification and carapace recognition tasks.

### 2.4. YOLOv5-Seg+SE Model Construction

YOLOv5 and its derivatives provide superior recognition accuracy, enhanced detection speed, and robust real-time performance relative to conventional object detection algorithms [33]. Compared with YOLOv5, YOLOv5-seg was improved in the following ways to make it particularly suitable for this task: (1) integration of a segmentation head for dual detection-segmentation capability; (2) improvement of the loss function with a segmentation component; and (3) implementation of multi-task training protocols to optimize task-weight balancing and inference efficiency. Therefore, to ensure that sex and integrity could be assessed and that the carapace could be detected in Chinese mitten crabs, YOLOv5-seg was used as the base model, and the SE attention module was used to develop a dedicated detection-segmentation algorithm that prioritized both accuracy and computational efficiency.

**Figure 4:**
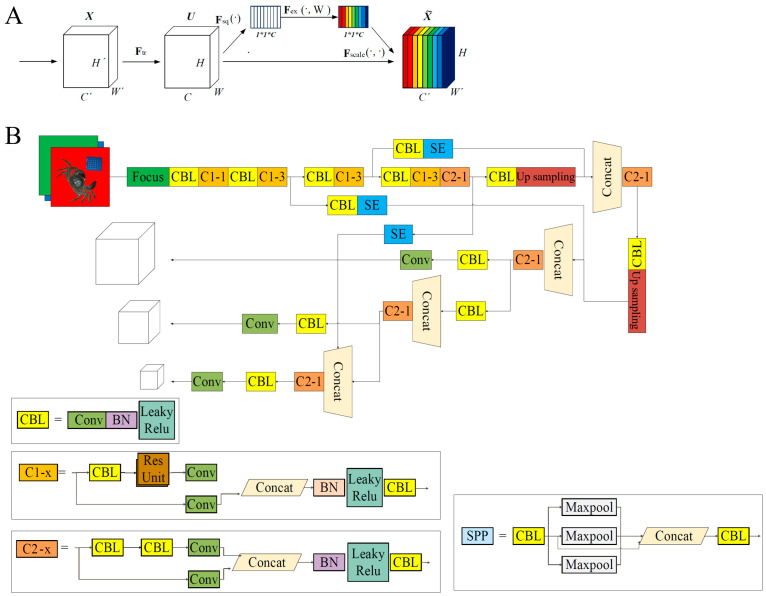
The model network. (**A**) Structure of the SENet module; (**B**) structure of YOLOv5-Seg+SE. Conv, convolutional layer; BN, batch normalization; CBL, Conv-BN-LeakyReLU; SE, squeeze-and-excitation; Concat, concatenation layer.

The SE attention mechanism improves model efficiency by learning adaptive channel-wise weights, allowing the model to focus more on informative feature channels [34]. As illustrated in Figure 4A, the structure operates as follows. First, a squeeze operation performs global pooling on each feature map, compressing it into a 1 × 1 × C descriptor (Figure 4A). Subsequently, an excitation operation learns channel-specific weights to adaptively recalibrate feature channel importance. Notably, this module preserves the spatial dimensions of input feature maps while enhancing channel-wise discriminative power. The resultant network structure of YOLOv5-seg+SE is shown in Figure 4B. Briefly, the network begins with a Focus layer and CBL blocks (Conv-BN-LeakyReLU) for down-sampling and feature extraction. SE modules dynamically recalibrate channel weights to emphasize informative features. The segmentation head employs up-sampling, concat operations, and SPP (Spatial Pyramid Pooling) to merge multi-scale context information for precise mask prediction. Key components such as residual connections (C2_x, Res Unit) and hierarchical design ensure efficiency and accuracy, which makes them suitable for the tasks in this study, such as crab integrity detection. The architecture balances detection and segmentation by optimizing both spatial and channel-wise features.

### 2.5. Morphological Feature Extraction

**Figure 5:**
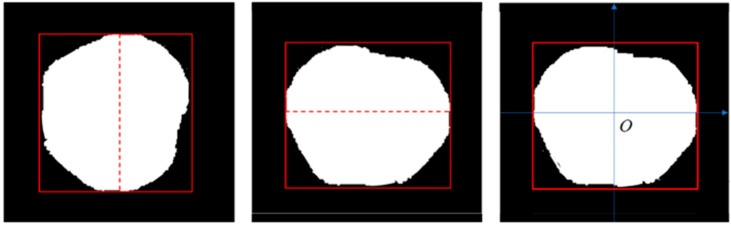
Adjustment of oblique angles and calibration of coordinates. The segmented carapace image is rotated to correct for oblique angles using template matching, which is established using the geometric center of the carapace, enabling accurate measurement of carapace dimensions.

The carapace dimensions of Chinese mitten crabs serve as an essential reference for their classification. The study utilizes the YOLOv5s-seg+SE algorithm for detecting and segmenting the crab’s carapace. Image processing is then used on the segmentation results to determine the carapace’s length, width, and area. This investigation employs an enhanced YOLOv5s-seg architecture incorporating SE attention modules for simultaneous carapace detection and precise segmentation in crab specimens. The resulting high-fidelity segmentation masks undergo subsequent morphometric analysis to computationally derive three key biometric parameters, including maximum carapace length, maximum width, and projected area. The detailed procedure is outlined as follows:

The YOLOv5s-seg+SE detection algorithm extracts carapace images, which are then corrected for tilt. Tilt correction was performed using a template-matching method to align the longest axis of the carapace horizontally. Following image binarization, the longest line of the crab’s carapace is used as a reference to align it parallel to the side boundaries of the carapace’s minimum enclosing rectangle, achieving tilt correction of the carapace contour image (Figure 5). Additionally, alignment results were visually verified during preprocessing, and errors were minimized through controlled image acquisition and calibration.

The image coordinate system was calibrated by first performing planar tilt correction and then establishing the reference frame using the geometric centroid of the segmented carapace as the origin (0, 0). The orthogonal axes were mathematically defined through the following transformations:

Ox = (Xmax + Xmin)/2(2)

Oy = (Ymax + Ymin)/2(3)

In this context, Ox denotes the *x*-axis origin, while Xmax and Xmin refer to the carapace contour image’s maximum and minimum *x*-axis coordinates, respectively. Similarly, Oy represents the origin of the *y*-axis, and Ymax and Ymin are the maximum and minimum *y*-axis coordinates of the carapace contour image.

Morphometric calibration was performed using a standardized reference board to establish the pixel-to-metric conversion ratio. The carapace dimensions were then quantified by the following: (1) counting the occupied pixels in the segmented binary image and (2) converting these values to physical measurements using the predetermined calibration coefficients. This approach yielded three principal biometric parameters: maximum length (L) along the anterior-posterior axis, maximum width (W) along the medial-lateral axis, and total projected area (A) of the carapace. The carapace’s actual area could be determined using the following formula:

A = S_0_N_0_ (4)

In the equation, A is the projected carapace area of the Chinese mitten crab (cm^2^), N_0_ represents the pixel count covering the carapace, and S_0_ indicates the area per pixel (cm^2^). The total pixel count representing the carapace’s length and width is derived from the processed image’s pixel data. The crab’s carapace length and width can be determined using the following formulas:

L_l_ = L_1_L_a_ (5)

L_w_ = L_2_L_b_ (6)

In the formulas, L_l_ and L_w_ refer to the actual carapace length and width of the crab in centimeters, respectively. L_1_ and L_2_ denote the pixel counts for the carapace’s length and width, while L_a_ and L_b_ represent the dimensions of a single pixel in length and width.

### 2.6. Evaluation of Model Performance

The model’s performance was assessed using standard YOLOv5 metrics: precision (P), recall (R), and mean average precision (mAP). The metrics are calculated using the following formulas:

P = TP/(TP + FP)(7)

R = TP/(TP + FN)(8)

mAP = (Σ^k^_i_ = 1APi)/k (9)

Here, TP denotes true positives, FP represents false positives, FN indicates false negatives, N stands for the total number of morphological classes, and APi refers to average precision for class i, calculated as the area under the precision-recall curve for that specific category.

## 3. Results

### 3.1. Sample Statistics

Each crab was assigned an identification number, showing its origin, year, and sex documented. Table 1 presents the statistics of the collected sample panel.

### 3.2. Model Optimization

This research develops a YOLOv5s-seg-based algorithm to detect sex and carapace features in Chinese mitten crabs. The model was trained using a mini-batch size of 16 samples per iteration, with hyperparameters initialized as follows: base learning rate (η) = 0.01, L2 regularization coefficient (λ) = 0.0005, and momentum (μ) = 0.937. Adaptive optimization strategies, guided by empirical tuning, were employed throughout the training to enhance detection accuracy. The full hyperparameter configuration is provided in Table 2.

A comprehensive comparative analysis was conducted to evaluate the YOLOv5s-seg+SE architecture against three benchmark models: YOLOv8-seg, YOLOv5l-seg, and YOLOv5n-seg. The evaluation employed identical experimental conditions, including the following: (1) standardized hyperparameter configurations, (2) uniform training/validation/test splits, and (3) consistent evaluation metrics, with detailed comparative results presented in Table 3. The precision and mAP were 0.74% and 1.1% higher for YOLOv5s-seg+SE, respectively, than for YOLOv8-seg; in addition, the recall of YOLOv5s-seg+SE was 3.7% higher than that of YOLOv8-seg. The precision, recall, and mAP were 2.14%, 1.9%, and 3.6% higher for YOLOv5s-seg+SE than for YOLOv51-seg, respectively. The precision, mAP, and recall were 10.84%, 13.4%, and 8.9% higher, respectively, for YOLOv5s-seg+SE than for YOLOv5n-seg. The YOLOv5s-seg+SE model demonstrated exceptional performance metrics, achieving 99.44% precision (P), 100% recall (R), and a mAP of 99.50%. A comparative analysis revealed that this architecture maintained superior computational efficiency, exhibiting 23% faster inference times than YOLOv5l-seg and requiring fewer training iterations to converge compared to YOLOv8-seg.

The superior performance of the YOLOv5-seg+SE model might be primarily due to the synergistic effect of segmentation capabilities and channel attention mechanisms. Compared to standard YOLOv5, which outputs bounding boxes, YOLOv5-seg adds a dedicated segmentation head, allowing the model to perform instance-level mask predictions that improve the precision of morphological measurements such as carapace area and length [35]. Additionally, the SE module enhances feature representation by adaptively recalibrating channel-wise responses based on global context [34]. This improves the model’s ability to capture fine-grained features and suppress irrelevant background noise, which is particularly beneficial in complex visual scenes such as varied crab postures or lighting conditions. These enhancements collectively contribute to the observed gains in precision, recall, and mAP.

### 3.3. Results of Integrity Identification

**Figure 6:**
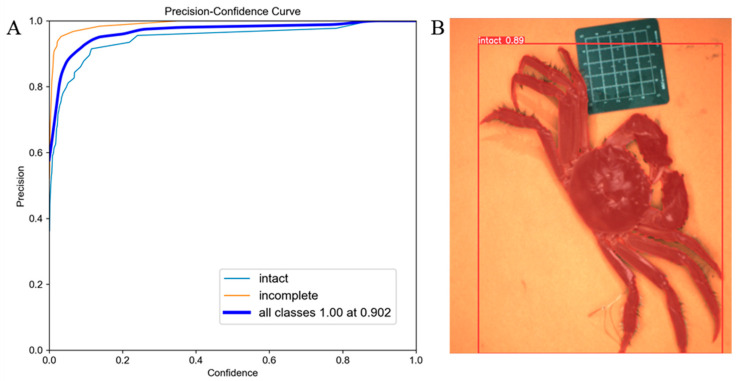
Integrity identification of Chinese mitten crab. (**A**) Precision-confidence curve of the sex identification model. (**B**) Integrity identification.

The detection and segmentation results of crab legs using YOLOv5s-seg+SE are shown in Figure 6. Figure 6A illustrates the precision-confidence curve of the integrity detection and segmentation process, and Figure 6B illustrates the effectiveness of integrity detection and segmentation for Chinese mitten crabs.

To validate the accuracy of the integrity detection for Chinese mitten crabs, we conducted integrity detection experiments on 115 images from the test set, and the results are presented in Table 4. The average accuracy of crab integrity detection reached 93.10%, with a mean recognition speed of 78.9 ms per image. This detection method aims to significantly enhance the production efficiency and quality control in crab processing. It should be noted that while the integrity detection method achieved high accuracy, a few challenges remain. In particular, crabs with minor limb damage, such as partial fractures or regenerated limbs, may still appear visually intact and be classified as such by the model. These borderline cases can be difficult to distinguish even during manual inspection. Additionally, overlapping limbs or occlusion in images may occasionally obscure missing appendages, leading to misclassification. Improving image angles and incorporating depth or multi-view data may help address these limitations in future work.

### 3.4. Sex Detection

**Figure 7:**
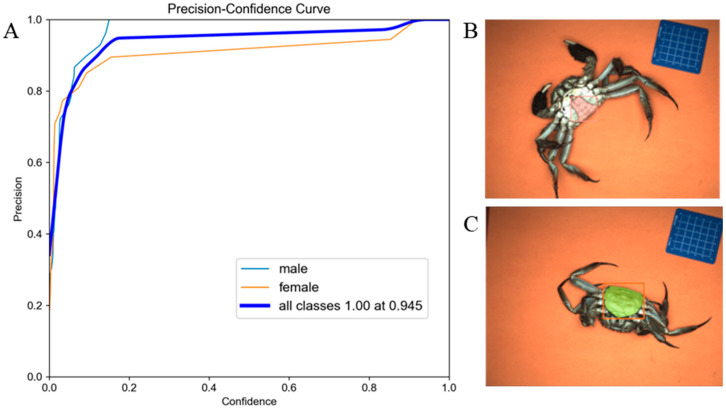
The performance of sex discrimination model of Chinese mitten crab. (**A**) Precision-confidence curve of the sex identification model. (**B**) Male recognition. (**C**) Female recognition.

The sexual dimorphism classification models were developed using the YOLOv5s-seg+SE architecture trained on abdominal morphology datasets. Figure 7 illustrates the model performance metrics of the sex detection model. Figure 7A presents the precision-confidence curve for the detection and segmentation of the abdominal area, and Figure 7B,C show exemplar detection outputs demonstrating the distinct umbilical morphologies characteristic of male (triangular) versus female (semicircular) of crab specimens, respectively.

We evaluated sex identification accuracy using a test dataset of 115 images, with results detailed in Table 5. The sex detection accuracy for Chinese mitten crabs achieved 100%, with an average image recognition speed of 74.9 ms. The high performance of the constructed model might be due to the distinct morphological differences between male and female abdominal flaps, pointed in males and rounded in females. These strong and distinct features, combined with high-quality annotations and sufficient training data, enabled the model to learn highly discriminative features with minimal ambiguity.

This detection system aims to automate the sex identification of Chinese mitten crabs, thereby lowering classification labor costs. Manual sex identification was conducted by 3 trained technicians based on the morphology of the abdominal umbilical region, where males display a narrow, pointed flap and females exhibit a broad, rounded flap. This method is commonly used in aquaculture and is generally considered reliable, with the reported accuracy above 95% in prior studies [35]. In this study, no discrepancies were found among the 300 manually labeled samples. However, occasional challenges arose, such as dealing with crab movement. These were minimized through careful handling and imaging, ensuring reliable ground truth for model training.

### 3.5. Prediction of Carapace Area and Size

**Figure 8:**
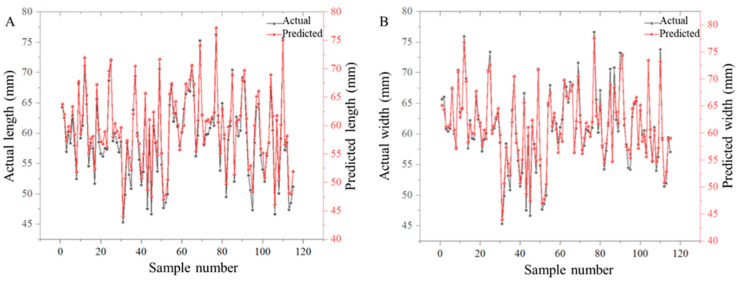
Performance of carapace size extraction. (**A**) Performance of carapace length prediction; (**B**) performance of carapace width prediction.

Accurate carapace morphometrics represent a critical determinant in crab quality grading. This automated assessment pipeline employed the following: (1) YOLOv5s-seg for precise carapace segmentation, followed by (2) computational extraction of three key biometric parameters (maximum length, width, and projected area). Method validation was conducted through the comparison between the output predictions and the manual caliper measurements for model performance (Figure 8).

The model was validated on an independent dataset of 115 crab specimens (73 males, 42 females). Exceptional measurement accuracy was achieved with a MAPE of 1.5% for carapace area segmentation and 1.3% and 1.7% for length and width detection, respectively, which fall well within acceptable limits for commercial grading. These minor discrepancies are unlikely to affect fatness classification or market valuation, confirming the model’s practical reliability. A strong linear correlation (R^2^ = 0.99, *p* < 0.001) was observed between automated predictions and manual ImageJ measurements, demonstrating the reliability of this approach for industrial grading applications. These results indicate that the computer vision system achieves less than 2% measurement errors (Figure 8) while processing crabs at 23 specimens per minute, representing a significant improvement over manual methods in both precision and throughput. The demonstrated accuracy in extracting critical morphometric parameters (carapace length, width, and area) establishes this automated approach as a robust solution for quality control in crustacean aquaculture operations.

### 3.6. Grading of Chinese Mitten Crab Based on Calculated Fatness

According to the first “Chinese Mitten Crab Product Grading Standard” [36], the criteria for grading male and female crabs based on the fatness level are as follows.

For male crabs, Grade I: >65%; Grade II: 62–65%; Grade III: 58–62%; and Grade IV: <58%.

For female crabs, Grade I: fatness level > 58%, Grade II: 55–58%; Grade III: 51–54%; and Grade IV: <51%.

To validate the method, 10 male and 10 female crabs were randomly selected, and the calculated measurements of the carapace area, fatness level, and fatness grading were compared with manual measurements. The experimental results are shown in Table 6.

As shown in Table 6, the methods for calculating the carapace area and fatness level, as well as the fatness grading approach, provide reliable data supporting the accuracy of the grading of Chinese mitten crabs. The algorithm successfully categorized all crabs into four grades, and these were perfectly consistent with the manual grading results. Discrepancies between automated and manual measurements were minimal, with a maximum absolute error of 0.92 cm and a maximum relative error of 3.1%; this demonstrated the high precision of the YOLOv5-seg-based segmentation. A comparison between the experimental and manual methods showed that the errors in carapace area and fatness level calculations were minimal, and both methods yielded consistent fatness grading results. The fatness level calculation errors in this study were within an acceptable range, and the performance of the fatness grading method was sufficiently high. Minor deviations in carapace area predictions may be attributed to slight inaccuracies in locating carapace spines during segmentation. Despite this, fatness calculation errors remained within acceptable limits, and the grading consistency confirmed the method’s reliability. Here, we employed the first “Chinese Mitten Crab Product Grading Standard” [36], which reflects market demand and directly impacts pricing, with higher-grade crabs commanding premium prices. By using both morphological data and projected area in fatness calculations, our grading method aligns closely with the commercial standards, ensuring its practical applicability in the crab industry. Therefore, this approach provides a robust technical foundation for the development of automated crab grading systems and meets industry requirements for efficiency and accuracy. Overall, our work provides a technical foundation for the development of automated grading equipment for Chinese mitten crabs.

## 4. Discussion

### 4.1. Rapid Extraction of Grading-Related Features

Grading and sorting are essential for increasing the economic value of agricultural products [37]. Recent advancements in intelligent sensing technologies, particularly machine vision systems, multimodal sensor arrays, and artificial intelligence algorithms, have significantly accelerated the progress in automated aquaculture monitoring and quality assessment [38,39]. While existing research has extensively explored intelligent grading systems for fruit and vegetable products, such as apples [40], citrus [41], and strawberries [42], similar applications for aquatic products remain understudied, particularly in automated quality assessment and sorting. Current grading practices for Chinese mitten crabs rely heavily on subjective manual evaluation of sexual characteristics and morphometric parameters [2,43]. This conventional approach introduces significant inter-rater variability, resulting in inconsistent quality assessments that fail to meet the precision and efficiency demands of modern aquaculture operations.

Machine-vision-based grading technology can achieve higher efficiency and accuracy compared with traditional manual grading methods [44]. Zhu et al. proposed an automated system for simultaneous sex classification and weight estimation of bundled crabs, achieving 95% accuracy in gender identification and maintaining less than 2% mean absolute percentage error in weight measurements through integrated RGB imaging and weight sensors [45]. Zhou et al. established a machine-vision-based grading system incorporating multiple quality parameters, including sexual characteristics and hepatosomatic index (as a measure of fatness). Their model demonstrated robust performance, achieving 97.5% accuracy in sex classification and 97% precision in fatness estimation [46]. However, the presence of tying ropes may introduce systematic errors in both gravimetric and visual measurements, compromising data accuracy during the weight assessment and image acquisition processes. This study utilizes YOLOv5s-seg incorporating SE to segment carapace and detect key grading features of unbundled Chinese mitten crabs, including integrity, carapace size, and sex. By implementing computer vision algorithms, our system aids in addressing three critical industry challenges and demonstrates satisfying performance, including the following: classification consistency, faster processing speed, and lower operational costs compared to traditional manual sorting. The proposed integrated vision system enables simultaneous extraction of multiple physiological parameters critical for quality grading, which may result in a significant advancement in aquatic product processing technology, in terms of standardized quality control, enhanced production efficiency, and reduced human intervention in industrial crab sorting operations.

### 4.2. Machine-Vision-Based Grading of Chinese Mitten Crabs

The YOLOv5s-seg+SE model achieved an exceptional performance, with 99.44% precision, 100% recall, and 99.50% mAP under a lightweight design, outperforming comparative models such as YOLOv8-seg in both speed and accuracy. This demonstrates that a moderately simplified network structure can balance efficiency and precision in specific applications and avoid the performance degradation seen in overly minimalistic models such as YOLOv5n. The 100% accuracy in sex detection likely stems from the morphological differences between the pointed abdominal flaps of male crabs and the rounded flaps of females. The results of the proposed grading method, which is based on the carapace area and fatness, were highly consistent with those of manual assessments, and errors were within practical tolerance limits (e.g., maximum relative error of 3.1%), which confirms the suitability of our method for real-world implementation.

### 4.3. Limitations of This Study

Challenges in image acquisition, such as sub-optimal backgrounds or water stains on the carapace or background, occasionally reduce processing accuracy [27]. Future studies could mitigate these issues by testing diverse background colors, ensuring that the surfaces are dry during imaging, and comparing alternative deep learning algorithms to further refine segmentation precision. Although the precision of the fatness grading of our method was high, there were still minor errors, which potentially stemmed from the lack of training data covering varied crab orientations [47]. Subsequent work is needed to optimize algorithmic parameters and expand training datasets to enhance detection robustness. Moreover, additional features, such as the texture or color of the carapace, might be included to enhance the grading robustness and generalization. These features also possibly correlate with flavor and nutritional status.

## 5. Conclusions

We developed a machine vision and deep-learning-based method for the quality grading of Chinese mitten crabs. A custom image acquisition device was designed to capture images of the abdomen and carapace of crabs. The YOLOv5-seg+SE model was used to identify the sex from abdominal images and carapace regions for area measurements. An improved fatness calculation method was proposed to enable quality classification. Our key findings are detailed below.

(1) A modified condition factor K′ was developed, which outperformed traditional Fulton and Jones factors. The K-based regression achieved higher R2 values, which enhanced the fatness accuracy assessment.
(2) The YOLOv5-seg+SE model achieved 100% accuracy in sex identification and a 0.995 mAP for carapace segmentation, with an inference speed of 74.9 ms per image, meeting real-time monitoring requirements.
(3) By integrating weight, sex, and carapace area, the proposed fatness-based grading method was fully consistent with manual observations, which validates its practical utility.

This approach will significantly enhance grading efficiency in the crab industry and reduce labor costs and product loss. The rapid inference speed of YOLOv5-seg+SE could allow it to be effectively used in automated online grading systems and advance intelligent detection capabilities in aquaculture. This methodology not only provides a technical foundation for crab grading but could also be modified for grading the quality of other crustaceans and agricultural products where morphological features determine quality. In future work, crabs from different regions and batches will be collected to improve model generalization and explore refined grading strategies by incorporating features such as cheliped ratios and shell coloration. Generally, our findings might facilitate the development of grading systems and devices for the Chinese mitten crab industry.

## Figures and Tables

**Table 1:** Statistics of the collected Chinese mitten crabs.

Sex	Number	Weight (g)	Width of Carapace (mm)	Length of Carapace (mm)
Male	150	90.06–250.04	48.64–82.14	48.32–78.55
Female	150	85.05–218.36	50.31–77.66	45.00–79.07

**Table 2:** Model’s super parameters.

Parameters	Value
Momentum (μ)	0.937
Learning_rate (η)	0.01
Epoch	300
Batch_size	16
Threshold value	0.5
L2 regularization coefficient (λ)	0.0005

**Table 3:** Performance comparison of YOLO models.

Models	P (%)	mAP (%)	Recall (%)
YOLOv8-seg	98.7	98.4	96.3
YOLOv5l-seg	97.3	97.6	96.4
YOLOv5n-seg	88.6	86.1	91.1
YOLOv5-seg+SE	99.44	99.50	100

**Table 4:** Results of crab integrity detection based on testing dataset.

Integrity	Confusion Matrix		Test Accuracy	Average Accuracy
	Intact	Incomplete		
Intact	65	5	92.86%	93.10%
Incomplete	3	42	93.33%	

**Table 5:** Results of crab sex identification.

Sex	Confusion Matrix		Test Accuracy	Average Accuracy
	Male	Female		
Male	73	0	100%	100%
Female	0	42	100%	

**Table 6:** Comparison of crab grading between manual estimation and fatness prediction.

	Sample Index	Experimental Method			Manual Methods		
		Carapace Area (cm^2^)	Fatness (K) (%)	Grade	Carapace Area (cm^2^)	Fatness (K) (%)	Grade
Male	M-1	30.37	75.27	I	30.28	75.93	I
	M-2	38.74	62.04	II	38.38	63.00	II
	M-3	32.00	56.28	IV	32.47	55.43	IV
	M-4	42.76	64.94	II	43.40	64.34	II
	M-5	47.78	61.14	III	48.13	61.14	III
	M-6	42.54	43.19	IV	43.01	42.81	IV
	M-7	44.24	61.90	III	43.57	61.90	III
	M-8	35.03	55.96	IV	35.66	57.11	IV
	M-9	41.08	63.19	II	40.39	62.41	II
	M-10	29.92	59.54	III	30.59	58.67	III
Female	F-1	37.44	56.73	II	37.09	57.34	II
	F-2	41.56	47.96	IV	41.67	47.22	IV
	F-3	59.58	50.82	IV	59.15	50.33	IV
	F-4	31.40	62.18	I	31.52	62.18	I
	F-5	36.55	63.26	I	36.21	64.70	I
	F-6	28.46	58.10	I	29.38	58.10	I
	F-7	29.93	55.38	II	30.60	55.85	II
	F-8	31.45	46.06	IV	30.62	45.48	IV
	F-9	31.73	48.09	IV	32.47	47.45	IV
	F-10	37.91	52.83	III	37.03	52.41	III

## Data Availability

The original contributions presented in this study are included in the article/Supplementary Materials. Further inquiries can be directed to the corresponding author.

## Supplementary Materials

The following supporting information can be downloaded at: https://www.mdpi.com/article/10.3390/foods14111989/s1, Supplementary File S1: Deduction of Improved Fatness Calculation.
