# Supplementary material for: Efficient and Non-Invasive Grading of Chinese Mitten Crab Based on Fatness Estimated by Combing Machine Vision and Deep Learning
Source: Foods. 2025 Jun 5;14(11):1989. doi: 10.3390/foods14111989 (PMC12154455; doi:10.3390/foods14111989)
Supplement: Supplementary file 1 [file foods-14-01989-s001.zip › foods-3644327-supplementary.pdf]

Deduction of Improved Fatness Calculation

Three condition factors were evaluated for fatness assessment:

Fulton condition factor K:

$$K = \frac{W}{L^3} 100\% \tag{1}$$

Jones condition factor B:

$$B = \frac{W}{HL^2} \tag{2}$$

In this research, the weight of crab, carapace length and carapace thickness of head and chest plate were combined with the density calculation formula to improve the formula. The condition factors were as follows:

Improved condition factor $K^{'}$:

$$K^{'} = \frac{3W}{AH} \tag{3}$$

Where W = body weight (g), L = carapace length (cm), H = carapace thickness (cm), and A = carapace area (cm²).

Calculate the K, B and $K^{'}$ values of each crab, and then calculate the average value of each condition factor, namely $\overline{K}$, $\bar{B}$ and $\overline{K^{'}}$. Use these average values to calculate the weight of each crab according to the following formula, that was, the inversed weight W_f.

$$W_{f,K} = \bar{K}L^3 \tag{4}$$

$$W_{f,B} = \bar{B}HL^2 \tag{5}$$

$$W_{f,K^{'}} = \frac{\overline{K^{'}}AH}{3} \tag{6}$$

To evaluate the accuracy of condition factors, linear regression analysis was conducted between actual weights and inversed weights. The slopes of regression lines were compared against the ideal value of 1.0 (representing the perfect fit $y=x$), combined with the coefficient of determination R^2, to assess the reliability of each condition factor for fatness. Additionally, the calculated K, B and $K^{'}$values for individual crabs were normalized by dividing them by their respective mean values $\overline{K}$, $\bar{B}$ and $\overline{K^{'}}$. These normalized ratios were then linearly fitted against actual weights to establish regression equations. A t-test was employed to evaluate deviations of regression slopes from 0 and intercepts from 1.0, thereby determining the relative performance of each condition factor.

As shown in Figure 1, the regression line derived from the modified condition factor $K^{'}$ aligns most closely with the ideal $y=x$ line, while those from Jones' (B) and Fulton's (K) factors

exhibit notable deviations. Table 1 summarizes the regression statistics between actual and inverted weights. All three condition factors demonstrate regression slope errors within 0.1, with K' achieving the smallest deviation (slope = 0.99121), indicating superior accuracy.

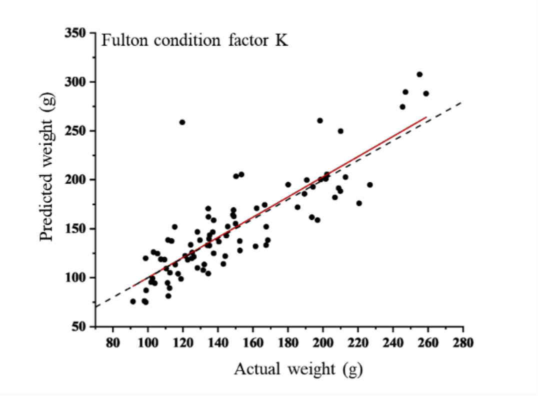

（a）condition factor K

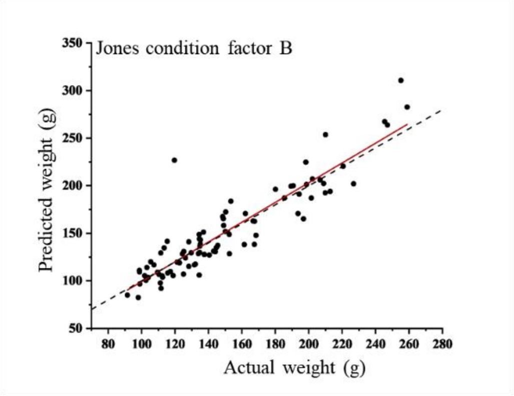

（b）condition factor B

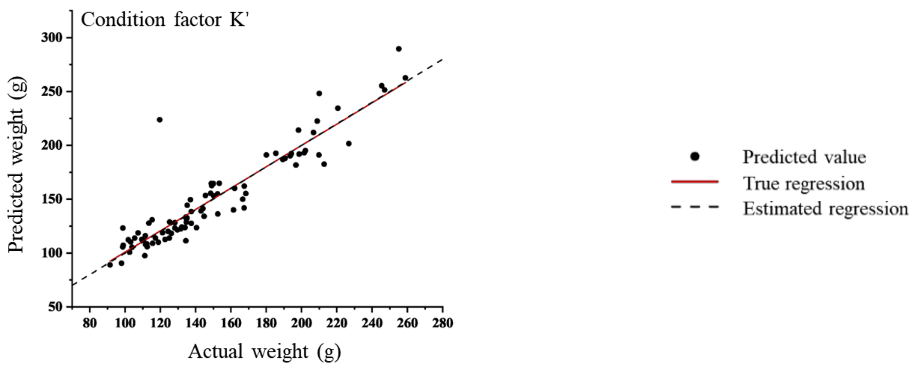

（c）condition factor K'

Figure S1 Regression curve of each condition factor

Table S1 Data comparison of each condition factor

Condition factor	slope	intercept	Residual sum of squares	R^2	Adjusted R^2
K	1.03142	-3.0359	63777.12532	0.70844	0.70501
B	1.04063	4.87195	33161.22633	0.8263	0.82425
K′	0.99121	1.71148	23534.51464	0.85878	0.85712

Figure S2 demonstrates that the regression slopes of $K/\bar{K}$, $B/\bar{B}$, $K'/\overline{K'}$, and $K'/\overline{K'}$ against actual weights are all close to 0. According to **Table S2**, the slopes for $K'/\overline{K'}$ and $B/\bar{B}$ are marginally closer to 0 compared to $K/\bar{K}$. However, when evaluating deviations of regression intercepts from the ideal value of 1.0, $K'/\overline{K'}$ exhibits the smallest deviation, while $K/\bar{K}$, $B/\bar{B}$ show deviations of approximately 0.2. Consequently, the accuracy ranking of

fatness metrics is $\overline{K'}$, $\bar{B}$ and $\bar{K}$.

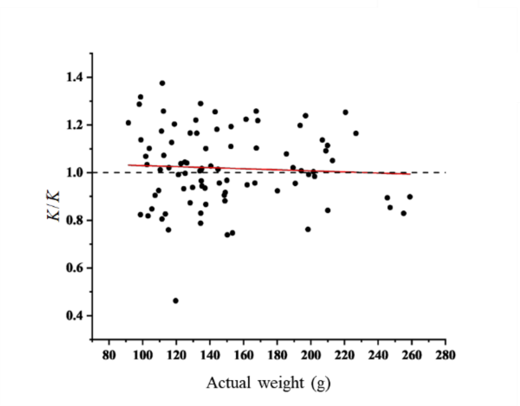

（a）$K/\bar{K}$ regression

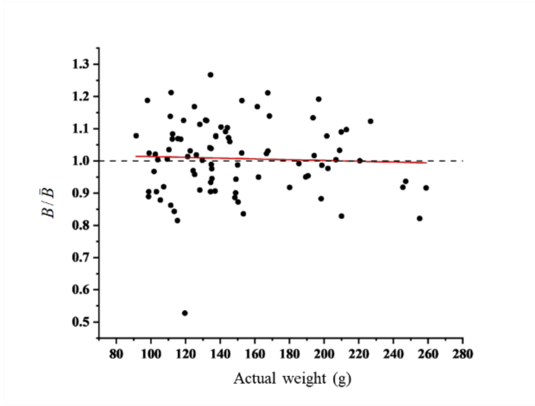

（b）$B/\bar{B}$ regression

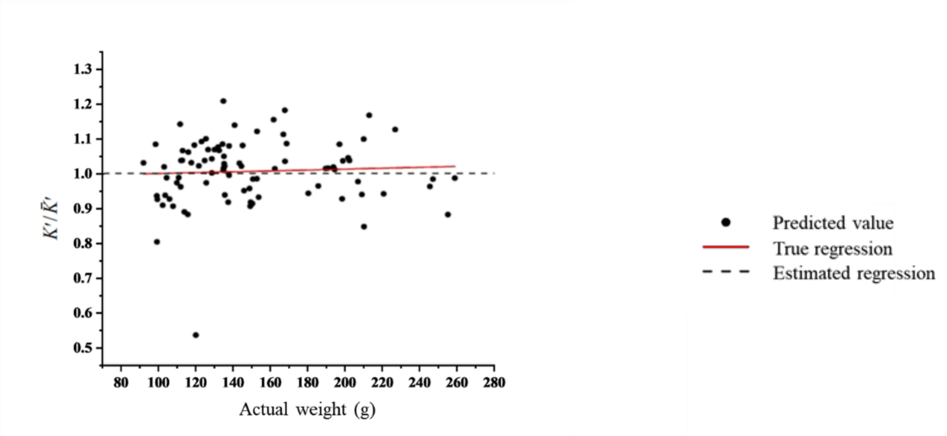

（c）$K'/\overline{K'}$ regression

Figure S2 Regression curve of $K/\bar{K}$, $B/\bar{B}$, $K'/\overline{K'}$

Table S2 Data comparison of $K/\bar{K}$, $B/\bar{B}$, $K'/\overline{K'}$

Condition factor	slope	intercept	Residual sum of squares	R^2	Adjusted R^2
$K/\bar{K}$	-0.0002	1.05170	2.26137	0.00315	-0.00857
$B/\bar{B}$	-0.0001	1.02569	1.12911	0.00192	-0.00983
$K'/\overline{K'}$	0.0001	0.98636	0.74882	0.00324	-0.00849

Thus, the improved condition factor K' achieves the highest accuracy for fatness evaluation in Chinese mitten crabs, outperforming both Fulton's (K) and Jones' (B) factors. Therefore, this study adopts K' as the optimal formula for fatness calculation.
